# The Dish-I-Wish open dataset on food preferences and parental influence

**DOI:** 10.3389/fnut.2026.1741707

**Published:** 2026-04-24

**Authors:** Anna Radygina, Yulia Zaripova, Daria Semenova, Sofya Kulikova

**Affiliations:** Centre for Cognitive Neuroscience, National Research University Higher School of Economics, Perm, Russia

**Keywords:** children, eating behavior, food preferences, nutrition, open dataset

## 1 Introduction

Over the past few decades, the academic community has been actively studying the problem of eating disorders in children and adolescents. It has become clear that these disorders can manifest themselves not only in adults, but also in children and young people. The consequences for physical health, personality development, social adaptation and overall quality of life necessitates a comprehensive approach to the prevention, diagnosis and treatment of these conditions.

Contemporary authors conceptualize this as a heterogeneous group of mental disorders, including anorexia, bulimia, overeating, and obesity. They focus on identifying the biological, psychological, and social factors that influence children's eating behavior (1). Biological aspects include genetic predisposition, metabolic characteristics, and central nervous system functioning. Psychological factors encompass impaired self-esteem, stressful situations experienced by the child, emotional disorders and trauma. Social factors under consideration may be subdivided into two categories: national trends and local characteristics. First, the increase in cases of eating disorders among children and adolescents is inevitably linked to external circumstances, such as the increased availability of a wide variety of food products and accelerated changes in household lifestyles during the period of globalization. However, the researchers also highlight the pivotal role of the immediate environment, family upbringing, and the inculcation of cultural and social norms in shaping children's eating habits. While eating disorders have historically been associated with adolescence, recent studies have identified the emergence of initial symptoms in preschool and early school-age children (2). These findings highlight the need for a comprehensive investigation of eating behaviors among children under the age of 10 years old. Unlike the trends observed among adolescents, social pressure from peers and public opinion has virtually no influence on the formation of eating habits in this age cohort. At the same time, parenting style and parents' eating habits have a significant impact (3).

Discourse among authors in the post-20th century has focused on investigating the relationship between parental behavior and the development of children's eating habits in early age groups. While parents are undoubtedly responsible for the structuring of their child's food environment, pediatricians have noted that children should be capable of determining portion sizes and regulating their calorie intake. This will contribute to the future prevention of overeating and other eating disorders in the younger generation (4–6). Large portions of high-calorie foods have been demonstrated to lead to excessive calorie consumption by children as young as 2 years old (7, 8). Forcible feeding, which disregards the child's genuine nutritional requirements, has a detrimental effect on their dietary preferences and appetite regulation (9). However, the existing research on the influence of parental behavior on the formation of a child's eating habits does not comprehensively describe the mechanisms of this phenomenon or fully elucidate the causal links between parents' organization of their child's nutrition and children's eating behavior (10, 11).

The academic community is faced with the following questions that require resolution. The central issue is whether children have the ability to control their own portion sizes, and if so, at what age they should be given the opportunity to develop healthy eating habits. This work presents a database that may help to investigate the age-related changes in the eating behavior of modern children, and suggest whether parents should exert control over this process.

Understanding children's food choices is therefore one of the key objectives of nutrition science. This approach will facilitate the establishment of risk groups and the identification of the specific factors influencing the development of pathological eating behavior. The problem of collecting reliable information about children's eating habits stems from the limitations of the survey and interview methods. One limitation is related to the subjectivity of responses and the desire of respondents to provide socially desired answers n. The majority of studies on children's eating behavior involve interviewing parents rather than children (12, 13). This phenomenon indicates that contemporary perceptions of children's eating habits are insufficient. In order to address this issue, new interactive data collection methods adapted for use by young children are required. Using gamification could be an effective solution. Such a paradigm allows children to engage in the research process, giving upon them a sense of autonomy, and providing them with the opportunity to recreate real-life scenarios involving food consumption at different mealtimes. The proposed methodology does not restrict children's decision-making, thereby giving the younger generation the opportunity to express their own preferences when planning their daily diet and to describe their parents' typical behavior when organizing family meals.

## 2 Methods

### 2.1 Participants

The study focuses on children aged 4–14 years (113 female, 90 male), which is a critical period for the development of dietary preferences. During this time, children are highly susceptible to familial, social, and environmental influences (14–16). This age range captures the emergence of basic decision-making capacities at age 4, through to the transition out of elementary school at age 11, after which peer influences predominate. The final sample included 203 children, with data collected in kindergartens and schools during the summer holidays to offer a representative insight into the development of eating behaviors.

**Recruitment and Parental Involvement:** respondents were recruited via a social media call. School principals, teachers, and kindergarten teachers helped to disseminate the information about the study to parents. Before collecting data from the children, parents were asked to sign a voluntary informed consent form for their child's participation, and they were given the opportunity to ask any questions about the data collection procedure. The experiment itself took place at the children's educational institution (school/kindergarten computer lab or a separate room). Parents were not present in the room during the data collection to avoid influencing the children's choices.

**Instructions and Procedure:** after receiving parental consent, the study organizers scheduled the experiment dates and reserved the computer labs where the experiments took place. Two or three instructors from the research team were present throughout the entire data collection session to instruct the children on the experiment procedure and answer any questions they had. For preschoolers (ages 3–6), the experiments were conducted individually. A research instructor (a 29-year-old woman with a teaching background and experience of working with children) met each child, opened the app, and explained each step of the experiment verbally in a native language, using simple, age-appropriate vocabulary. The interface itself was visually intuitive (clicking images, using a slider), and all in-app content (food names, labels) was fully translated and presented into the native language. The platform was localized for the study.

**Duration:** the experimental session for each child lasted approximately 15–20 min.

**Language:** the platform and all instructions for the participants were given in the native language (Russian). The examples and descriptions in this paper are English translations of the original materials used in the study.

### 2.2 Research instrument

The study uses a custom web application called Dish-I-Wish as its primary research instrument. Designed with age-appropriate UX/UI principles (17, 18), it features a gamified, visually intuitive interface that enables children to select and adjust the portion sizes of foods and beverages using vivid imagery and large interactive elements with minimal text. A complementary parent module collects data on family dietary habits and socio-environmental influences on children's eating behaviors.

### 2.3 Data collection and procedures

Data were collected between May 19 and June 30, 2025.

The study utilizes a two-stage, family-centered data collection protocol implemented through the Dish-I-Wish web application to capture both children's food preferences and their perceptions of parental dietary norms.

#### 2.3.1 Stage 1: parental questionnaire

Parents provide demographic and child-specific information (e.g., age, gender, dietary restrictions) and report on household eating practices (e.g., home cooking, restaurant use).

#### 2.3.2 Stage 2: child interactive session

Following parental input, the child engages with a gamified interface in two sequential tasks:

1. Free selection: the child constructs preferred meals (breakfast, lunch, dinner), freely choosing foods and portion sizes as they would for themselves, without any restrictions.

2. Perceived norms selection: the child repeats the task, selecting meals “as my parents would give me,” thereby indicating their perception of parental feeding practices.

This design allows us to make direct comparisons between autonomous preferences and perceived parental norms, offering insight into individual choice and familial influence interact in children's eating behavior.

**Figure 1:**
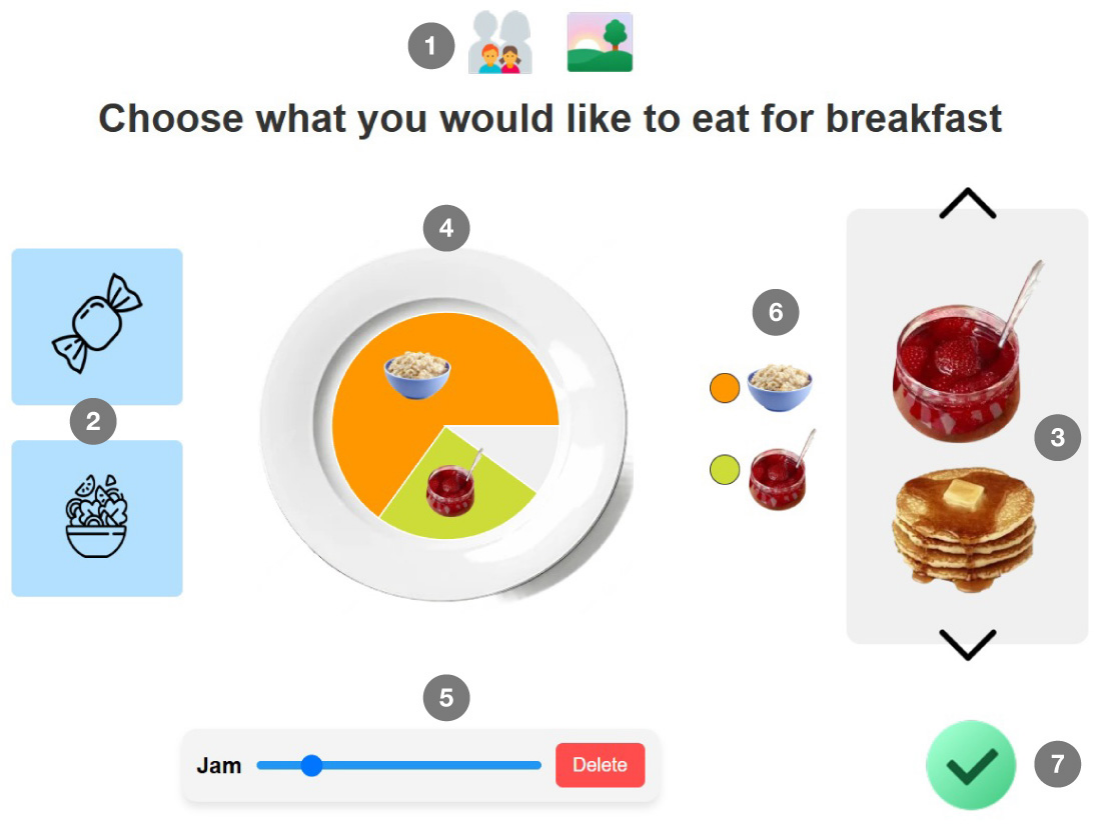
Example interface from the *Dish-I-Wish* web application: breakfast food selection screen. Numbered annotations identify key interactive elements: (1) Selection mode indicators: left icon toggles respondent perspective (child vs. parent); right icon selects meal type (breakfast, lunch, or dinner). Icons made by Icons8.com. (2) Dish category filters: items are grouped into thematic subcategories (e.g., “sweet” and “non-sweet” for breakfast). Icons made by Freepik from www.flaticon.com. (3) Dish carousel: a vertically scrollable list of visually representative dish images. (4) Virtual plate visualization: selected dishes appear as color-coded segments, with arc lengths proportional to portion size. (5) Portion adjustment slider: allows fine-tuning of the portion for the currently selected dish. (6) Selected dish icons: thumbnail previews summarizing previously chosen items. (7) Confirmation button: finalizes the meal configuration and proceeds to the next task stage.

An example of the meal-selection interface is shown in Figure 1.

## 3 Sample characteristics and dataset overview

The dataset comprises anthropometric and metabolic measurements taken from a sample of respondents. Descriptive statistics for each variable are presented in Table 1.

**Table 1:** Descriptive statistics of children characteristics.

Age group	Variable	Mean	Std. dev.	Min	Max
**Prescool**	weight	19.72	4.2	15	32
	height	1.14	0.08	1.05	1.35
	age	5.08	0.81	4	6
	EER	1,486	165.26	1,301.95	2,060.1
	BMI	15.04	1.53	13.22	18.44
**Primary school**	Weight	31.66	7.93	21	56
	Height	1.38	0.08	1.2	1.59
	Age	8.65	1.04	7	10
	EER	1,771.77	223.17	1,434.8	2,550.13
	BMI	16.53	2.79	12.07	24.24
**Middle school**	weight	45.88	11.98	25	80
	Height	1.56	0.1	1.35	1.83
	Age	12.09	0.91	11	14
	EER	2,094.45	364.3	1,546.03	3,271.97
	BMI	18.61	3.63	12.73	29.62

Here Weight is the respondent's body mass, measured in kilograms (kg); Height is the respondent's standing body height, measured in meters (m); Age is the respondent's age at the time of measurement, reported in complete years; Estimated Energy Requirement (EER) is the calculated daily dietary energy intake estimated to maintain energy balance, measured in kilocalories (kcal); and Body Mass Index (BMI) is a derived measure of weight-for-height, calculated as weight in kilograms divided by the square of height in meters (kg/m^2^). Anthropometric measurements of height and weight were obtained from respondents immediately prior to the assessment. BMI was calculated using the standard formula (kg/m^2^) from measured height and weight. EER values were estimated using the equations recommended by the Institute of Medicine (19), adjusted for age, sex, and physical activity level (assumed to be moderate for all participants, as were data collected during the summer holidays in educational settings and a physical activity coefficient set to 1.13).

The distribution of BMI in our sample (mean = 17.39 kg/m^2^, range: 12.07–29.62) falls within the expected range for Russian school-aged children, consistent with national epidemiological studies (20) and World Health Organization (21) growth reference percentiles.

In addition, the dataset includes parent-reported information on the child's diagnosed allergies, appetite level, and usual household food preparation practices (e.g., home cooking, food delivery, or dining out), and family income level.

To illustrate how the collected data can be leveraged to examine children's eating behavior, Table 2 shows the average values for the caloric and macronutrient content of meals self-selected by the children in the study.

**Table 2:** Descriptive statistics of meals chosen by children for themselves.

Age group	Meal type	Total calories (mean)	Total calories (std. dev.)	Number of products (mean)	Number of products (std. dev.)
**Pre-school**	Breakfast	683.75	338.69	4.36	1.52
	Lunch	703.19	405.32	7.12	3.02
	Dinner	825.04	350.19	6.88	2.68
	**Total per day**	**2,211.97**	**662.48**		
**Primary school**	Breakfast	546.71	296.3	4.49	1.92
	Lunch	636.11	295.03	7.73	3.31
	Dinner	767.32	406.71	6.8	3.17
	**Total per day**	**1,950.15**	**773.80**		
**Middle school**	Breakfast	512.26	285.53	4.33	1.38
	Lunch	609.76	307.32	7	2.53
	Dinner	675.24	349.04	5.99	2.54
	**Total per day**	**1,797.26**	**740.06**		

The table shows the total (for three meals) calorie values for children's age categories in bold.

All values reported in Table 2, such as mass, total calories, total carbohydrates, total protein, and total fat, correspond to the portion of food and beverages selected by each child during the interactive session. These values are not based on a standardized unit, such as 100 g. These absolute nutrient amounts were estimated by integrating two inputs: (1) the percentage of the plate area occupied by a food item (as chosen by the child via the interface) and (2) the physical density of that food item (in g/cm3), which was obtained from authoritative food composition databases or measured empirically where needed.

To convert visual plate coverage into physical mass, we assumed a total edible plate volume of 300 ml for solid meals and 200 ml for beverages. For example, if a child selected “chocolate cake” to occupy 30% of the plate and the cake's density was 0.6 g/cm3, the resulting mass would be approximately 54 g. Absolute macronutrient and caloric values were then calculated using food composition tables. This approach enables the dataset to reflect the realistic meal compositions constructed by children, which are relevant for behavioral and public health analyses.

Density values were primarily sourced from the Russian National Food Composition Database (22) and cross-verified with USDA FoodData Central (23). For items lacking published density data, density was determined empirically using the hydrostatic (water displacement) method. Specifically, for each such food item for which density data was lacking, we prepared three standard portions under controlled kitchen conditions, weighed each portion (in grams), and measured its volume by water displacement using a graduated cylinder. The density was then calculated as the ratio of the mean mass to the mean volume across the three replicates. All measurements were conducted at room temperature (22 ± 2 °C), and the results were rounded to two decimal places. This empirically derived density was subsequently used to convert children's visual plate selections into estimated physical masses in the dataset.

A complete data dictionary detailing all variables is provided in the Supplementary material (File Data_Dictionary.md).

## 4 Conclusion

The released dataset includes the following: detailed anthropometric characteristics of participants (weight, height, age, BMI, estimated energy requirements) alongside comprehensive nutritional profiles of selected food items (caloric content, macronutrient composition, portion sizes, and visual plate representation). The descriptive statistics presented in Tables 1, 2 confirm the variability and representativeness of the sample and food stimuli, thus supporting its utility for a variety of analytical purposes.

This dataset enables researchers to investigate the complex interplay between children's internal preferences, their perception of external parental control, their anthropometric characteristics and nutritional outcomes, moving beyond simple dietary recall. This dataset allows for the exploration of various key research questions. For instance, researchers can examine how children's autonomous food choices differ from what they perceive their parents would serve them. They can also investigate whether the discrepancy between a child's own preference and their perceived parental norm varies by age, gender, or body mass index. Another critical line of inquiry involves comparing the portion sizes that children select for themselves with the portions that they believe their parents would give them, particularly across different food categories such as vegetables vs. sweets. Furthermore, the dataset supports the analysis of how children's food choices evolve with age, specifically whether meals become more calorically dense or nutritionally varied, and whether this trend differs between girls and boys. The influence of the family environment can be studied by correlating parent-reported household practices, such as the frequency of home cooking and dining out, with children's actual and perceived choices.

Additionally, the relationship between a child's perception of their parents' portion norms and their own BMI or EER can be assessed. From a nutritional standpoint, the dataset enables the nutritional quality of meals constructed by children based on their own preferences to be evaluated, including average calories and macronutrient balance, and how these compare to age-appropriate dietary guidelines. Pattern analysis can identify distinct clusters of children based on their autonomous meal composition profiles. Researchers can also determine whether specific food items or categories predict a high total caloric intake in a child-constructed meal, and whether the variety of foods selected relates to the meal's overall nutritional density or calorie count. Finally, the data can be used to build predictive models, exploring whether features from the meal selection data, such as total calories chosen, fat preference, or vegetable avoidance, can help to identify children who are potentially at risk of developing suboptimal eating patterns.

By making these data openly accessible, we aim to facilitate further research into the early determinants of eating behavior, support the development of child-adapted dietary assessment tools, and encourage interdisciplinary work at the intersection of nutrition science, developmental psychology, human–computer interaction, and public health. Future venues of research could include exploration of patterns of food choice, test hypotheses about familial influence, or develop predictive models of dietary risk by developing machine learning models trained on this dataset.

## Data Availability

The datasets presented in this study can be found in online repositories here - Figshare doi: 10.6084/m9.figshare.30454502.

## References

[1] Barakat S McLean SA Bryant E Le A Marks P National Eating Disorder Research Consortium. Risk factors for eating disorders: findings from a rapid review. J Eat Disord. (2023) 11:8. doi: 10.1186/s40337-022-00717-4 36650572 PMC9847054

[2] Chatoor I. Eating disorders in infancy and early childhood. In: The Oxford Handbook of Child and Adolescent Eating Disorders: Developmental perspectives. Oxford library of psychology. New York, NY: Oxford University Press (2012). p. 198–213

[3] Larsen JK Hermans RCJ Sleddens EFC Engels RCME Fisher JO Kremers SPJ. How parental dietary behavior and food parenting practices affect children's dietary behavior. Interacting sources of influence? Appetite. (2015) 89:246–57. doi: 10.1016/j.appet.2015.02.012 25681294

[4] Stein AD Shea S Basch CE Contento IR Zybert P. Variability and tracking of nutrient intakes of preschool children based on multiple administrations of the 24-hour dietary recall. Am J Epidemiol. (1991) 134:1427–37. doi: 10.1093/oxfordjournals.aje.a116048 1776617

[5] Mela DJ. Determinants of food choice: relationships with obesity and weight control. Obes Res. (2001) 9:249S−55. doi: 10.1038/oby.2001.127 11707550

[6] Spill MK Birch LL Roe LS Rolls BJ. Serving large portions of vegetable soup at the start of a meal affected children's energy and vegetable intake. Appetite. (2011) 57:213–9. doi: 10.1016/j.appet.2011.04.024 21596073 PMC3140700

[7] Satter E. Feeding dynamics: helping children to eat well. J Pediatr Health Care. (1995) 9:178–84. doi: 10.1016/S0891-5245(05)80033-1 7629684

[8] Johnson SL. Improving preschoolers' self-regulation of energy intake. Pediatrics. (2000) 106:1429–35. doi: 10.1542/peds.106.6.1429 11099599

[9] Daniels LA. Feeding practices and parenting: a pathway to child health and family happiness. Ann Nutr Metab. (2019) 74:29–42. doi: 10.1159/000499145 31234189

[10] Pimpin L Ambrosini GL Llewellyn CH Johnson L van Jaarsveld CHM Jebb SA . Dietary intake of young twins: nature or nurture? Am J Clin Nutr. (2013) 98:1326–34. doi: 10.3945/ajcn.113.065250 24047917 PMC3798084

[11] Mahmood L Flores-Barrantes P Moreno LA Manios Y Gonzalez-Gil EM. The influence of parental dietary behaviors and practices on children's eating habits. Nutrients. (2021) 13:1138. doi: 10.3390/nu13041138 33808337 PMC8067332

[12] Alves-Santos NH Castro IRR de Anjos LAD Lacerda EM de A Normando P Freitas MB de . General methodological aspects in the Brazilian national survey on child nutrition (ENANI-2019): a population-based household survey. Cad Saude Publica (2021) 37:e00300020. doi: 10.1590/0102-311x00300020 34495099

[13] Blaine RE Kachurak A Davison KK Klabunde R Fisher JO. Food parenting and child snacking: a systematic review. Int J Behav Nutr Phys Act. (2017) 14:146. doi: 10.1186/s12966-017-0593-9 29096640 PMC5668962

[14] Nicklaus S. Development of food variety in children. Appetite. (2009) 52:253–5. doi: 10.1016/j.appet.2008.09.018 18940212

[15] Savage JS Fisher JO Birch LL. Parental influence on eating behavior: conception to adolescence. J Law Med Ethics. (2007) 35:22–34. doi: 10.1111/j.1748-720X.2007.00111.x 17341215 PMC2531152

[16] Patrick H Nicklas TA. A review of family and social determinants of children's eating patterns and diet quality. J Am Coll Nutr. (2005) 24:83–92. doi: 10.1080/07315724.2005.10719448 15798074

[17] Aparna KS. Top 10 UI/UX design tips for child-friendly interfaces. Aufait UX (2024) Available online at: https://www.aufaitux.com/blog/ui-ux-designing-for-children/ (Accessed June 6, 2025)

[18] Golden Rules of Interface Design for Children. Available online at: https://www.drimlike.com/en/blog/10-golden-rules-of-interface-design-for.html (Accessed June 6, 2025)

[19] Standing Standing Committee on the Scientific Evaluation of Dietary Reference Intakes Subcommittee Subcommittee on Interpretation Uses Uses of Dietary Reference Intakes Subcommittee Subcommittee on Upper Reference Levels of Nutrients Panel Panel on the Definition of Dietary Fiber Panel Panel on Macronutrients. Dietary Reference Intakes for Energy, Carbohydrate, Fiber, Fat, Fatty Acids, Cholesterol, Protein, and Amino Acids. National Academies Press (2005).

[20] Martinchik AN Laikam KE Kozyreva NA Mikhaylov NA Keshabyants EE Baturin AK . Prevalence of overweight and obesity in children. Problems of Nutrition. (2022) 91:64–72. doi: 10.33029/0042-8833-2022-91-3-64-72 35852979

[21] World Health Organization. The WHO Child Growth Standards. Geneva: WHO. Available online at: https://www.who.int/tools/child-growth-standards (Accessed November 6, 2025)

[22] Skurikhin IM Tutelian VA. Tables of the Chemical Composition and Caloric Content of Russian Food Products. Moscow: DeLu Print. (2007) p. 275.

[23] USDA. Food Data Central. Washington D. C: USDA. Available online at: https://fdc.nal.usda.gov/ (Accessed November 7, 2025)

