# Supplementary material for: The Dish-I-Wish open dataset on food preferences and parental influence
Source: Front Nutr. 2026 Apr 24;13:1741707. doi: 10.3389/fnut.2026.1741707 (PMC13154275; doi:10.3389/fnut.2026.1741707)
Supplement: Supplementary file 1 [file Table_1.docx]

# Dish-I-Wish: A Dataset on Children’s Food Choices, Portion Sizes, and Meal Context

## Overview

This dataset contains information about meals consumed by children, including details about food type, portion sizes, nutrients, and caregiver-reported characteristics (appetite, meal preparation habits, household income, etc.).

Data were collected in 2025, Perm, Russia.

## Data Dictionary

| **Column Name** | **Type** | **Units / Allowed Values** | **Description** |
| --- | --- | --- | --- |
| child_id | string | Alphanumeric identifier (e.g., “CH001”) | Unique identifier for each child participant. |
| meal_type | categorical | breakfast, lunch, lunchDessert, dinner, dinnerDessert | Type of meal recorded. lunchDessert, dinnerDessert is dessert consumed as part of the lunch / dinner meal. |
| actor | categorical | parent, child | The role: how the child would choose meals himself or how would his parent choose for him. |
| name | string | Free text (e.g., milk, apple) | Name of the consumed food item. |
| calories | number | kcal per 100 g | Energy content of the food item per 100 grams. |
| carbon | number | grams per 100 g | Carbohydrate content per 100 grams of the food item. |
| protein | number | grams per 100 g | Protein content per 100 grams of the food item. |
| fat | number | grams per 100 g | Fat content per 100 grams of the food item. |
| density | number | g/cm³ | Physical density of the food item — mass per unit volume, measured in grams per cubic centimeter (g/cm³). Reflects how compact the food is as a physical substance (e.g., milk ≈ 1.03 g/cm³, bread ≈ 0.29 g/cm³). |
| mass | number | grams | The actual mass (weight) of the portion consumed is calculated from the percentage of the product on a plate and its physical density. |
| total_calories | number | kcal | Total calories consumed from this item (calories × mass / 100). |
| total_carbon | number | grams | Total carbohydrates consumed from this item. |
| total_protein | number | grams | Total protein consumed from this item. |
| total_fat | number | grams | Total fat consumed from this item. |
| type | categorical | beverage, food | Food group or category of the item. |
| percentage | number | Percent (0 – 100) | The percentage of the plate area occupied by this item. |
| gender | categorical | male, female | Child’s gender. |
| age | number | years | Child’s age in full years at the time of data collection. |
| height | number | centimeters | Child’s height. |
| weight | number | kilograms | Child’s weight. |
| allergy | string | Free text; e.g., milk, peanuts, gluten | Known food allergies reported by caregiver. Use “NaN” if no allergies. |
| appetite | categorical | 1, 2, 3, 4  1 - Poor appetite: Child often experiences a lack of desire to eat and may skip meals.  2 - Fluctuating appetite: Appetite varies significantly from days of overeating to days of very low food consumption.  3 - Moderate appetite: Child eats adequately but occasionally refuses meals.  4 - Excellent appetite: The child consistently demonstrates enjoyment of meals and shows a positive receptiveness to new dishes. | Caregiver-reported general appetite level of the child. |
| meal_prep | categorical | 1, 2, 3, 4  1 - We usually cook meals at home—someone in the family does the cooking.  2 - We alternate: sometimes we cook at home, sometimes we order food.  3 - We eat at home but order delivery from restaurants.  4 - We often eat out and prefer dining at cafés and restaurants. | Caregiver-reported typical pattern of meal preparation or sourcing. |
| incomes | categorical | 1, 2, 3, 4  1 - We live very frugally, there’s enough for daily expenses, but buying clothes is already difficult.  2 - We can afford food and clothing, but purchasing major household appliances is problematic.  3 - Overall, we are financially secure, but we cannot afford expensive purchases without taking out a loan or using savings.  4 - We are well-off and can fairly easily afford major purchases such as a car or an expensive vacation. | Caregiver-reported household income level category. |
| is_hungry | boolean | true / false | Whether the child reported or appeared hungry before this meal. |
| BMI | number | kg/m² | Body Mass Index, calculated as weight (kg) / (height (m))². |
| EER | number | kcal/day | Estimated Energy Requirement — daily caloric need based on age, sex, weight, height, and activity level. |
| age_group | categorical | preschool (4–6 ages), primary school(7–10 ages), middle school (11–14 ages) | Variable indicating the educational stage-based age cohort of the participant |
